# Effects of Fucoidan Powder Combined with Mineral Trioxide Aggregate as a Direct Pulp-Capping Material

**DOI:** 10.3390/polym14122315

**Published:** 2022-06-08

**Authors:** Mijoo Kim, Marc Hayashi, Bo Yu, Thomas K. Lee, Reuben H. Kim, Deuk-Won Jo

**Affiliations:** 1 UCLA School of Dentistry Restorative Materials and Applied Dental Research Laboratory, Los Angeles, CA 90095, USA; vagusmj@gmail.com (M.K.); mhayashi@dentistry.ucla.edu (M.H.); boyu@dentistry.ucla.edu (B.Y.); tlee@dentistry.ucla.edu (T.K.L.); rkim@dentistry.ucla.edu (R.H.K.); 2 Section of Restorative Dentistry, UCLA School of Dentistry, Los Angeles, CA 90095, USA; 3 Section of Dentistry, Department of Prosthodontics, Seoul National University Bundang Hospital, Seongnam 13620, Korea

**Keywords:** direct pulp capping, fucoidan, mineral trioxide aggregate, osteogenesis, portland cement, tooth regeneration

## Abstract

The development of direct pulp-capping materials with favorable biological and structural properties is an important goal in restorative dentistry. Fucoidan is a sulfated, fucose-containing polysaccharide obtained from brown seaweed, with a wide range of applications; however, its use as a direct pulp-capping material has not been examined. This study aimed to evaluate the mechanical, physical, and biological effects of fucoidan combined with conventional mineral trioxide aggregate (MTA) for direct pulp capping. The capping materials were created using Portland cement (80 wt%) and zirconium oxide (20 wt%) as base components, compared with base components plus 5 wt% fucoidan (PZF5) and base components plus 10 wt% fucoidan (PZF10). The initial and final setting time, compressive strength, chemical components, cell viability, adhesion, migration, osteogenesis, and gene expression were analyzed. Fucoidan significantly reduced the initial and final setting time, regardless of quantity. However, the compressive strength was lower for PZF5. Sulfur levels increased with fucoidan. The biological activity improved, especially in the PZF5 group. Cell migration, Alizarin Red S staining, and alkaline phosphatase activity were upregulated in the PZF5 group. Fucoidan is a useful regenerative additive for conventional pulp-capping materials because it reduces the setting time and improves cell migration and osteogenic ability.

## 1. Introduction

Restoring damaged teeth necessitates the removal of the compromised parts and coverage with artificial dental materials. Dental caries, one of the main pathologies requiring dental treatments, cause pulp exposure in up to 40% of clinical cases when deep lesions in the dentin are removed [1]. If the pulp is exposed unintentionally during caries removal and the remaining tooth structure is intact, an endodontic treatment, such as pulpectomy or pulpotomy, and direct pulp capping can be an effective treatment strategy. Compared with a root canal treatment, direct pulp capping is a minimally invasive procedure that saves time, costs, and effort for both clinicians and patients [2,3,4]. However, pulp vitality is sometimes maintained only for limited periods, and the pulp eventually requires root canal treatment due to irreversible pulpitis or pulp necrosis, depending on the capping materials and the patient’s age [2,5,6]. Therefore, developing capping materials that protect the exposed pulp and its highly regenerative ability is critical for maintaining pulp vitality.

Mineral trioxide aggregate (MTA) is a typical direct pulp-capping material; it induces an alkaline pH by leaching calcium hydroxide, kills the remaining bacteria within the capped area, and induces dentin bridge formation [7]. The exposed pulp is sealed more tightly using MTA than calcium hydroxide, and this material is highly stable, which is essential for the restorative success [8]. However, MTA has some disadvantages, including a long setting time, high cost, toxicity due to increased alkalinity, sandy consistency when mixing, and discoloration. Thus, modifying MTA to obtain an excellent dentin sealing and induce a biologically favorable pulp reaction is critical for a satisfactory clinical outcome. Several studies suggested possible MTA modifiers to reduce the setting time and enhance handling properties, such as a mixture of 1% methylcellulose and 2% CaCl_2_ [9], propylene glycol [10], 3% NaOCl gel [11], and polyvinyl alcohol [12]. However, an optimal balance between hard tissue regeneration and improved mechanical/physical characteristics has yet to be established.

Fucoidan, a sulfated polysaccharide, has been considered as a new additive to modify the properties of the original MTA. It is obtained from various species of brown seaweed and has a molecular structure and function similar to heparin. Moreover, like heparin/heparan sulfate, it is involved in cell proliferation and differentiation [13,14], including osteogenic differentiation [15,16,17,18]. It also has antitumoral, antiviral, and anti-inflammatory properties [19,20]. Fucoidan is used worldwide, especially in the food and pharmaceutical industry, and has promising therapeutic effects [21]. However, its use in dentistry is still limited. Therefore, this study aimed to evaluate the mechanical, physical, and biological properties of MTA modified with fucoidan as a direct pulp-capping material.

## 2. Materials and Methods

### 2.1. Experimental Materials

The base material of our experimental MTA was composed of white Portland cement (80 wt%; Union Cement, Seoul, Korea) and zirconium oxide ([ZrO_2_] 20 wt%; Sigma Aldrich, St. Louis, MO, USA). Fucoidan (a polysaccharide with a broad range of molecular weights) was purchased from Haewon Biotech (Seoul, Korea). It was composed of 61.5% polysaccharides and 23.5% sulfate and was extracted from the brown seaweed *Laminaria japonica*. Various concentrations of fucoidan (i.e., 0, 5 and 10 wt%) were added to the base material to obtain the PZ, PZF5, and PZF10 groups of samples, respectively. The water/powder ratio was 0.3, and the materials were mixed using a plastic spatula for 1 min. For biological comparisons, a commercial MTA was obtained from Maruchi (Wonju, Korea) and mixed according to the manufacturer’s instructions. The mixed materials were inserted into Teflon molds of different sizes (indicated in the sections below) and placed in a 37 °C water bath to simulate the oral environment.

### 2.2. Characterization of Direct Pulp-Capping Materials

#### 2.2.1. Setting Time

The components in each group were mixed and inserted into Teflon molds with an inner diameter of 10 mm and a height of 2 mm. The initial and final setting times for all samples were measured following the American Society for Testing and Materials (ASTM) C266-03 and International Organization for Standardization (ISO) 6876 regulations [22,23]. The Gilmore needle used to test the initial setting time (“initial needle”) had a weight of 100 g and an active tip diameter of 2.0 mm. The needle used for the final setting time (“second needle”) had a weight of 400 g and an active tip diameter of 1.0 mm. The initial needle was applied lightly to the surface of each sample. This procedure was repeated every 5 min for all materials until the needle did not create a complete circular depression on the specimen surface. For each sample, the time elapsed between the end of mixing and the unsuccessful indentation was recorded in minutes and defined as the “initial setting time.” The “final setting time” was determined following the same procedures using the second needle, with a 400 g load. Five sets of measurements were obtained for each material.

#### 2.2.2. Compressive Strength

The fabricated samples were 6 mm in diameter and 12 mm in height. The compressive strength of the specimens was determined according to the method recommended by ISO 9917-1 using a universal testing machine (Instron 5942; Instron Co., Norwood, MA, USA) [24]. The crosshead speed was 1 mm/min along the long axis. The specimens were maintained in a warm bath at 37 °C for 1 day and stored in a desiccator for 28 days. Five specimens were used for each estimate. The compressive strength was calculated using the following equation:

Compressive strength = 4 P/π D^2^, (1)

where P is the maximum load (in N), and D is the mean diameter of the specimen (in mm).

#### 2.2.3. Physical and Chemical Characteristics

The surface morphology of the experimental materials was examined under a field emission-scanning electron microscope (FE-SEM; S-4800; Hitachi, Tokyo, Japan). An energy dispersive X-ray (EDX) analysis was performed to determine the elemental composition of the materials, using the EDX-System (S-4800; Hitachi, Tokyo, Japan), attached to the FE-SEM instrument with an accelerating voltage of 10 kV. Fourier-transformed infrared spectroscopy (FT-IR) analysis was also performed (Nicolet iS10; Thermo Scientific, Waltham, MA, USA) in a frequency range of 4000–400 cm^−1^ to identify the functional groups of the materials.

### 2.3. Biological Evaluation of Direct Pulp-Capping Materials

The experimental specimens for biological evaluation were 10 mm in diameter and 5 mm in height. Each sample was added to a well containing the medium after the hardening process in a water bath. Human osteoblast-like cells (MG63) were grown in an α-minimal essential medium, supplemented with 10% fetal bovine serum, penicillin (100 units/mL), and streptomycin (100 mg/mL) at 37 °C in a humidified atmosphere of 5% CO_2_ and 95% air. Osteogenic differentiation was induced by replacing the medium with osteogenic medium (OM) containing 50 μg/mL l-ascorbic acid, 10 mM/mL β-glycerophosphate, and 10^−7^ M/mL dexamethasone. The medium was replaced every 2 days during the incubation period. The tests were performed three times independently.

#### 2.3.1. Cell Viability

The cells were seeded at a density of 1 × 10^4^ cells/well in a 24-well plate. After 24 h of adhesion, an experimental specimen was added to each well. The cell viability in each group was determined using a 3-(4,5-dimethylthiazol-2-yl)-2,5-diphenyltetrazolium bromide (MTT) assay on days 3 and 7. The optical density was measured using a microplate absorbance reader at a wavelength of 570 nm (Epoch; Biotek, Winooski, VT, USA).

#### 2.3.2. Cell Migration

Human umbilical vein endothelial cells (HUVECs) were seeded in triplicate in 6-well culture plates (2.5 × 10^4^ cells/well). After 24 h of culture, the cells reached over 90% confluency. A 1 mL autoclaved pipette tip was used to scratch the middle of each well floor. The cells were washed with phosphate-buffered saline (Gibco, Waltham, MA, USA) and conditioned with extracts from each experimental specimen (surface area/medium volume ratio: 3 cm^2^/mL, according to ISO 10993-12). Cell migration was examined using a microscope (Olympus, Tokyo, Japan) after 12 h culture periods.

#### 2.3.3. Osteogenic Differentiation Assays

For Alizarin Red S (ARS) staining, cells were fixed with 70% ethanol for 1 h at 25 °C. They were stained with 1% ARS (Sigma) for 10 min and subsequently washed with deionized water. Each well was treated with a 10% cetylpyridinium chloride in 10 mm sodium phosphate (pH 7.0) buffer for 15 min. Optical density was quantified using a microplate reader (Epoch) at 562 nm.

As an indicator of osteogenic differentiation, alkaline phosphatase (ALP) activity was determined using *p*-nitrophenyl phosphate (*p*NPP, 3 mM final concentration) as the substrate in 0.7 m 2-amino-methyl-1-propanol, pH 10.3, and 6.7 mm MgCl_2_. The *p*NPP working solution was added to each test plate (24 wells, 1 × 10^4^ cells/well) and incubated for 1 h. Optical density at 405 nm was obtained using an enzyme-linked immunosorbent assay (ELISA) reader.

### 2.4. Statistical Analysis

One-way analysis of variance was used (α = 5%) to determine significant differences between groups. Pair-wise multiple comparisons were performed using Tukey’s tests when significant differences emerged from the one-way analysis of variance.

## 3. Results

### 3.1. Mechanical and Chemical Properties of Direct Pulp-Capping Materials

**Figure 1:**
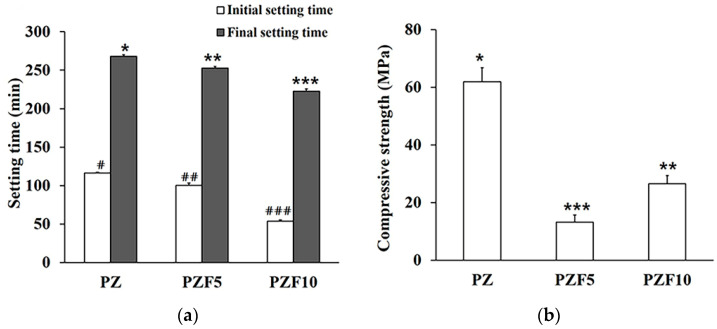
(**a**) Initial/final setting time and (**b**) compressive strength of the experimental groups: PZ (80 wt% of Portland cement and 20 wt% of zirconium oxide as base components), PZF5 (PZ plus 5 wt% of fucoidan), and PZF10 (PZ plus 10 wt% of fucoidan). *, **, *** indicates a significant difference between the groups with different fucoidan content (final setting time and compressive strength). #, ##, ### indicates a significant difference compared to the groups with different fucoidan content (initial setting time).

The initial and final setting times decreased significantly after the addition of fucoidan (*p* < 0.05), as shown in Figure 1a. In particular, PZF10 had an initial setting time > 50% shorter than the PZ time. However, the compressive strength was significantly lower in the PZF5 than in the PZ group (Figure 1b). Interestingly, PZF10 showed greater compressive strength than PZF5 (*p* < 0.05).

**Figure 2:**
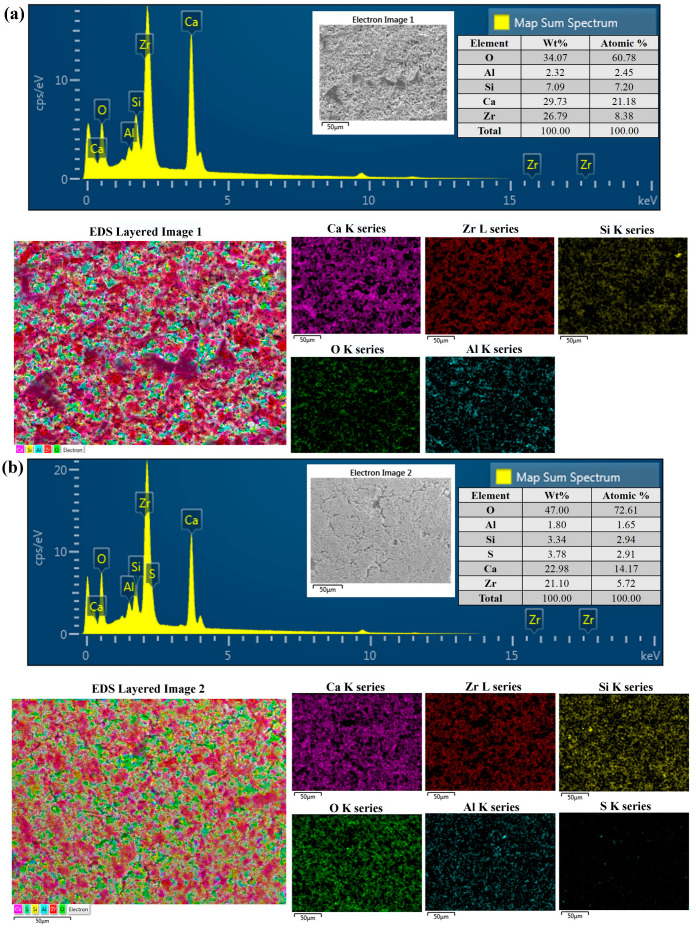

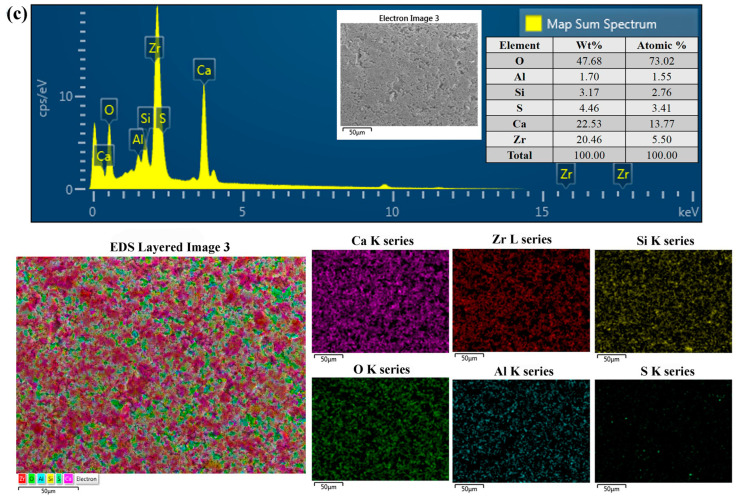
FE-SEM and EDX analysis of the experimental groups: (**a**) PZ, (**b**) PZF5, (**c**) PZF10. The surface morphology of PZ, PZF5, and PZF10 was studied under FE-SEM. The major components were analyzed and visualized with EDX. FES-SEM: field emission-scanning electron microscope; EDX: energy dispersive X-ray.

The FE-SEM results and chemical compositions are summarized in Figure 2. According to the surface images, the PZ group had rougher surfaces than the PZF5 and PZF10 groups. All groups had O, Al, Si, Ca and Zr as their components. In the surface analysis, PZ5F (Figure 2b) and PZ10F (Figure 2c) both contained sulfur (PZ5F, 3.78 wt%; PZ10F, 4.46 wt%).

**Figure 3:**
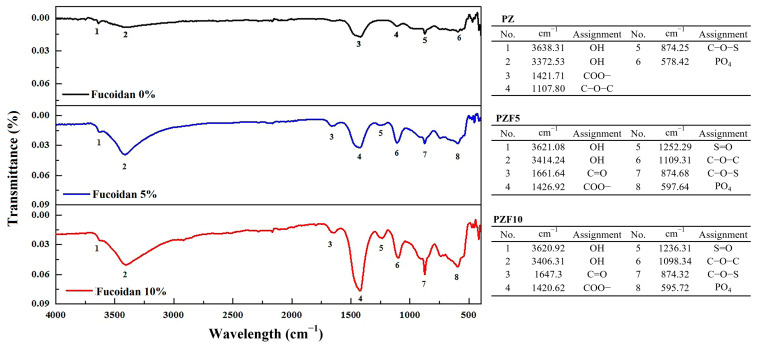
Fourier-transformed infrared spectroscopy results of PZ, PZF5, and PZF10. The functional groups in relation to the specific peaks are described in the text.

FT-IR was used to reveal the differences in chemical compositions between groups; the results of this analysis are shown in Figure 3. A peak at around 1420 cm^−1^ (COO-) increased in relation to the fucoidan content. The C–O–S bonds at 874 cm^−1^ were strengthened with fucoidan. PZ did not have the S=O stretching vibration; however, the spectra of PZF5 and PZF10 showed absorption bands of 1252 and 1236 cm^−1^ (S=O stretching vibration) and 597 and 595 cm^−1^ (related to the asymmetric bending vibration of the PO_4_ groups).

### 3.2. Biological Evaluation of Direct Pulp-Capping Materials

**Figure 4:**
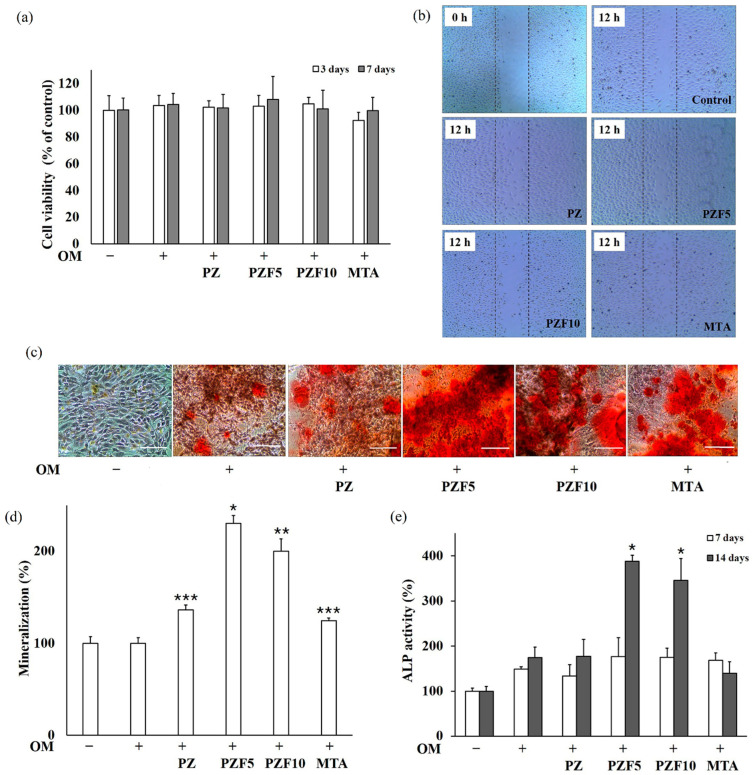
Biological effects of PZ, PZF5, PZF10 and MTA on cell viability, adhesion, and migration of MG63 human osteoblast-like cells. (**a**) Cell viability was determined by MTT assay after 3 and 7 days. (**b**) Cell migration was visualized by microscopy (×100) after 12 h. Alizarin Red S staining (ARS) was (**c**) visualized and (**d**) quantified at 612 nm. (**e**) The alkaline phosphatase (ALP) activity was analyzed by an ALP activity test and compared. Cells were cultured in osteogenic medium (OM) containing 50 μg/mL L-ascorbic acid, 10 mM/mL β-glycerophosphate, and 10^–7^ M/mL dexamethasone. * *p* < 0.05, ** *p* ≤ 0.01, *** *p* ≤ 0.001 compared to the group only conditioned by OM. The data are representative of three independent experiments. Scale Bar = 50 µm.

Cell viability, migration, and osteogenesis were compared between the test groups, as summarized in Figure 4. Cell viability was similar between groups after 3 and 7 days (*p* > 0.05). PZF5 exhibited a minor increase in cell viability after 7 days of culture compared with the other groups; however, this difference was not significant (Figure 4a). HUVECs conditioned with PZF5 exhibited a higher cell migration than that of the other groups after 12 h of culture (Figure 4b). Cells with 10% fucoidan (PZF10) migrated less than cells in the PZ, PZF5 and MTA groups and had a level of migration similar to the control group after 12 h.

ARS was visualized and quantified in the mineralization assay after 14 days of culture, as shown in Figure 4c,d. The optical density was highest in the PZF5 group, followed by the PZF10, PZ and MTA groups. As shown in Figure 4e, the ALP activity was higher in the PZF5 and PZF10 groups than in the others after 14 days, and the PZF5 group had the highest optical density values (*p* < 0.05).

## 4. Discussion

MTA is widely considered the gold standard for biocompatible and osteogenic direct/indirect pulp capping and apexogenesis of immature teeth; moreover, it can be used as an endodontic sealer. It mainly consists of Portland cement and radiopacifiers, and recent studies have described modifications of its components to enhance the mechanical and biological characteristics of the material [2,25,26,27]. This study used ZrO_2_ instead of bismuth oxide, a typical radiopacifier, as an MTA component to prevent discoloration and toxicity. ZrO_2_ (20 wt%) shows a radiopacity comparable to that of conventional MTA [28,29] and increases cell viability [30] via specific alterations in cell signaling pathways [31,32,33,34]. In addition, it does not exhibit genotoxicity in murine osteoblasts [35]. Thus, this investigation evaluated Portland cement and ZrO_2_ modified by various concentrations of fucoidan as a direct pulp-capping material.

Dental materials need to meet certain requirements for their clinical usage; for MTA, as a direct pulp-capping material, easy handling, a short initial setting time, and efficient regenerative ability are necessary. One disadvantage of the original MTA, poor handling, is caused by its sandy consistency after mixing it with water; the use of fucoidan could overcome this issue according to the current study. As fucoidan mixed with water becomes a viscous hydrocolloid fluid, MTA combined with fucoidan presents a doughy consistency, which improves the handling properties and facilitates its application on the exposed pulp. Previous studies showed that the addition of a hydrophilic synthetic polymer provided easier handling and anti-washout characteristics compared to the standard MTA [12,36]. Using a modified MTA with more favorable consistency, clinicians could decrease the application time within the tooth and reduce the waste of MTA powder. Adding fucoidan at a concentration > 10 wt% was not possible due to its high viscosity, preventing the mixing with Portland cement and ZrO_2_ in the pilot study. Therefore, the amount of fucoidan tested was set to 0, 5 and 10 wt% in the current study.

The initial setting time, including the loss of gloss, for PZF10 was less than half of the corresponding PZ time. We inferred that fucoidan added to Portland cement and ZrO_2_ resulted in a viscous fluid with increased reaction rates during setting procedures. While studies with 3 to 5% polyvinyl alcohol [36] and elastin-like polypeptide [37] showed an increased initial setting time, fucoidan accelerated the MTA hydration process. In contrast to other polymers, fucoidan is an excellent natural modifier, as it reduces the initial and final setting times and provides improved handling properties. For direct pulp capping in the clinics, the initial setting time is more critical than the final one because temporary restorations cover the exposed pulp area immediately after applying MTA in the dentin. Thus, a short initial setting time is beneficial for the primary stability of MTA. Fucoidan is advantageous, since it significantly decreases the initial MTA setting time and facilitates the mixture placement as an anti-washout gel, according to our study results.

The compressive strength significantly decreased after adding fucoidan compared to the original MTA mixture. However, previous studies investigating the MTA compressive strength in relation with several additives are controversial. MTA with polyvinyl alcohol showed a decreased compressive strength [36], whereas with elastin-like polypeptide [37] and nano-SiO_2_ [38], the strength increased. The different cross-linking structure modifies this property after the setting process of each material. However, a significant decrease in compressive strength for MTA mixed with fucoidan is not problematic, considering its clinical usage. In contrast, osteogenic and dentinogenic capabilities are more relevant for a direct pulp-capping material because a temporary seal is replaced with a permanent one once tertiary dentin is formed. Interestingly, 10% fucoidan demonstrated higher strength values than 5% fucoidan. These results were consistent with a previous study, in which increasing fucoidan weight fractions in PCL/fucoidan biocomposites improved the tensile strengths of the material by approximately 70%, whereas the strength at 10 wt% fucoidan abruptly worsened [39]. This result was attributed to the widely distributed and aggregated fucoidan powders, where the applied tensile stress could be highly concentrated. It is assumed that 10 wt% of fucoidan was also aggregated inside MTA and hampered the integrity of the materials.

FT-IR peaks differentiated the three experimental groups. The C–O–S bending vibration at 874 cm^−1^ was a unique characteristic attributed to the fucoidan sulfate [40]. Furthermore, the peak at 1250 cm^−1^ was caused by the S=O stretching vibration of the sulfate esters. SEM images and EDX mapping also showed sulfur, the main component of fucoidan, indicating that the fucoidan powders were effectively embedded. As the concentration of fucoidan increased, sulfur was more highly distributed in the Portland cement and ZrO_2_ combinations. However, some aggregated fucoidan powders were also found for high concentrations of fucoidan, especially PZF10. As a result of aggregation, stress on the fibers may have occurred under applied strain, resulting in improved mechanical properties for a high concentration of fucoidan.

According to this study, the addition of fucoidan powder gave a definitive advantage in terms of biological effects. While cell viability was not significantly different between the groups, cell migration was especially high in the PZF5 and MTA group. PZF10 showed a migration level similar to the control group after 12 h of culture. A previous study reported that an increased fucoidan concentration elevated the cell viability and proliferation to a certain level [41]; thus, 10 wt% of fucoidan can be considered a limit for increasing cell viability. The present experimental results suggest that 5 wt% fucoidan improves cell adhesion and proliferation-associated factors, and possibly cell-cycle-associated proteins, via the FAK and PI3K-Akt signaling pathways, which are related with this upregulation [42]. Mineralized granules increased significantly in the PZF5 group, and ALP activity was highest with the addition of fucoidan (PZF5 and PZF10) after 14 days. This result is a marked improvement compared to the commercially used MTA product, considered a gold standard direct pulp-capping material, and demonstrates the possibly strong positive effects of fucoidan combined with MTA for treating patients.

The modification of dental materials requires the consideration of pros and cons in terms of mechanical, physical, and biological effects. Materials for direct pulp capping necessitate fast setting times, firm adhesion to the adherent, and hard tissue regeneration induction in clinical usage, rather than mechanical strength. Therefore, this study demonstrated the possibility of clinical use of fucoidan powder as an MTA modifier for direct pulp capping. MTA with 5 wt% fucoidan is an especially good candidate, which might provide a rapid initial setting time and high biological activity in human dentin. This study only tested MTA with 0, 5 and 10 wt% fucoidan; hence, additional studies should examine a wider range of concentrations to maximize the effects of fucoidan powder as a biocompatible additive to the MTA in further study.

## 5. Conclusions

In conclusion, fucoidan is an effective additive for the traditional pulp-capping material MTA and has satisfactory properties, especially in terms of biological activity. Further studies considering the application of MTA to load-bearing areas are necessary to further assess a lower compressive strength than MTA alone.

## Data Availability

The data presented in this study are available on request from the corresponding author.
